# Genetic resistance of eight native Egyptian chicken breeds having chicken B-cell marker 6 gene post-challenge with field strain of Marek’s disease-induced tumor virus

**DOI:** 10.14202/vetworld.2018.1510-1515

**Published:** 2018-10-29

**Authors:** Hala A. Shaheen, H. A. Hussein, M. M. Elsafty, M. A. Shalaby

**Affiliations:** 1 Central Laboratory for Evaluation of Veterinary Biologics, Cairo, Egypt; 2 Department of Virology, Faculty of Veterinary Medicine, Cairo University, Giza, Egypt

**Keywords:** *ChB6* gene, Egyptian chicken breeds, Marek’s disease, polymerase chain reaction, transmission electron microscope

## Abstract

**Aim:**

The aim of this work was to detect chicken B-cell marker 6 (*ChB6*) gene in some native breeds in Egypt and find the relationship between founded genes in these different breeds to determine the resistance of native Egyptian breeds of chicken to Marek’s disease (MD).

**Materials and Methods:**

A total of 14 different chicken breeds (30 each) including ten native breeds in addition to SPF Lohmann, High Line, Bovans, and Roodiland were used. Blood samples were collected for the detection of (*ChB6*) by polymerase chain reaction (PCR) assay and sequenced to determine the presence or absence of *ChB*6 gene. Experimental infection was done using local field isolated MD virus (MDV) of 11 (1 day old) unvaccinated chick breeds having no maternal antibodies against MDV. Ten breeds of them carry *ChB6* gene, eight breeds were native, and the rest two breeds were SPF Lohmann and High Line in addition to a group of *ChB6* gene-lacking breed (Bovans) were infected. Spleen samples were collected from all infected breeds at 20^th^, 25^th^, 30^th^, 35^th^, and 40^th^ weeks post-infection and tested by PCR assay for the detection of MDV. Furthermore, at 40^th^ week post-infection, tumorized spleen sample of Bovans breed was collected and prepared for examination by transmission electron microscope (TEM) to confirm the presence of MDV.

**Results:**

Our results revealed the positivity of 10 out of 14 breeds (Gimmizah, Sinai, Dandarawi, Fayoumi, Golden Montazah, Matrouh, Beheri, Dokki, SPF Lohmann, and High Line) to the presence of *ChB6* gene and resistance to MDV infection, while the Bovans, Mandarah, Inshas and Roodiland breeds lack the *ChB6* gene and are susceptible to MDV infection. The collected spleen samples revealed negative for the presence of challenged MDV by PCR in 10 breeds (Gimmizah, Sinai, Dandarawi, Fayoumi, Golden Montazah, Matrouh, Beheri, Dokki, SPF Lohmann, and High Line) and positive for Bovans breed. TEM is used to confirm MDV infection in Bovans group which demonstrated tumors.

**Conclusion:**

The study confirms the relationship between the presence of *ChB6* gene in our native breeds and the absence of tumors.

## Introduction

Marek’s disease (MD) is a complex, immunosuppressive lymphomatous which induces T-lymphoma and neuropathic disease of domestic fowl caused by an alphaherpesvirus characterized by paralysis, chronic wasting, lymphoma development in the viscera and musculature, and blindness in chickens. MD symptoms vary in severity based on virus strain as well as bird genotype and vaccination status with death occurring in susceptible, non-immunized chickens. MD occurs at 3-4 weeks of age or older and is the most common between 12 and 30 weeks of age. Chickens may persistently infect with MD virus (MDV) without developing clinical disease. Diagnosis is made on clinical signs and gross/microscopic lesions.

Infection by MDV can be detected by virus isolation and the detection of viral antigen or antibodies. In chickens, tumors that resemble those produced by MDV have to be differentiated from avian retroviruses such as avian leukosis virus and reticuloendotheliosis virus [1-3]. However, successful vaccination control these diseases but losses still occur [4].

There are three lymphocyte surface antigens (Bu-1, Thy-1, and Ly-4) associated with the resistance against MDV [5]. The gene coding for Bu-1 has been isolated [6] and designated as chicken B-cell marker 6 (*ChB6*) which is highly glycosylated membrane protein expressed on chicken B lymphocytes through most of their development and shows a significant association with resistance. The mechanism of resistance was unknown in 1990, later on, they found that the *ChB6* as a B cell surface antigen induces a physiological signal for apoptosis in self-reactive lymphocytes, thus preventing autoimmune diseases in birds [7,8].

This study can enhance the breeding program of these breeds in Egypt. The present study was designated to investigate the *ChB6* gene presence and find the relationship between them in some different poultry breeds in Egypt through phylogram.

## Materials and Methods

### Ethical approval

Adequate measures were taken to minimize pain and animal discomfort. Experiments were carried out in accordance with the guidelines laid down by the International Animal Ethics Committee and in accordance with local laws and regulations.

### Chicken breeds

Fourteen different chicken breeds (30 each) including native breeds, namely Mandarah, Gimmizah, Sinai, Dandarawi, Inshas, Fayoumi, Golden Montazah, Matrouh, Beheri, and Dokki, SPF Lohmann, High Line, Bovans, and Roodiland. All groups were reared in separated isolators.

### Polymerase chain reaction (PCR) assay for detecting the presence of ChB6 gene in these 14 chicken breeds

Blood samples were collected in K_3_ EDTA tube vacutainer (VOMA MED^®^ lot No.150302) and then transported in ice-box to the laboratory, and the DNA extraction step was done for the detection of the resistant gene to MD (*ChB6*) by PCR assay.

Primers amplified a 215-bp fragment (BIOFLUX kit, cat No. BSC06S1.) covering exon 3 from genomic DNA. The PCR was performed in a total volume of 25 µL, containing 25 ng of genomic DNA, 0.8 µL of each oligonucleotide (nt) primer (F: 5’-GCTTCCCCAATGGAACTG-3’ and R: 5’ GAGCACAATGGGCCTAGTC-3’ and master mix kit MY Taq™ Red mix), 2.5 µL of 10×PCR reaction buffer, 1.5 mM MgCl2, 200 µL of each deoxynucleotide (dNTP), and 1 U of Taq DNA polymerase. Cycling parameters included initial cycle of 94°C for 5 min, then 35 cycles of 94°C for 1 min, 55°C for 1 min, and 72°C for 1 min, with a final extension step of 15 min at 72°C [9,10].

The electrophoreses of the PCR product were carried out using agarose (1%) of molecular biology grade containing ethidium bromide with a final concentration of 0.5 µg/ml at 75 V for 15 min in 1×TBE buffer, against GeneRulerTM100 bp Plus DNA Ladder (ferments). The expected product of corrected size (215 bp) was visualized and photographed using UV Transilluminator BioDoc Analyze Digital Systems (Biometra, Germany) and Polaroid films [9].

### Nt sequence of PCR products

The PCR products were subjected to sequencing. Amplified DNA band of the PCR product was excised and purified from the gel using QIAquick PCR Product Extraction Kit (Qiagen Inc., Valencia, CA) according to the manufacturer instruction. The purified PCR products were sequenced by Biosystems 3130 automated DNA sequencer (ABI, 3130, USA), and the data were analyzed by BioEdit software with similar sequence from GenBank National Center for Biotechnology Information (NCBI) [6,11]. Nt sequences of *ChB6* (accession number X92865) were used to search in the GenBank (NCBI) for nt and amino acid analysis [11].

Assembly of the consensus sequences and alignment trimming was performed with the laser gene DNASTAR group of programs (DNASTAR Inc., Madison, WI), using Clustal W method [11,12]. The gene alignment identity, divergence percentage, and phylogram were carried out and drawn using DNA star - MegAlign software [11-12-13].

A phylogenetic tree is comparing the relatedness of the sequence of the *ChB*6 gene in different positive chicken breeds with other similar *ChB*6 genes sequence from GenBank of the world using MEGA6 software [11,13].

### Experimental design

MDV local field isolate was isolated and characterized in 2015 from tumor samples of MD-infected flock. The virus was detected by PCR assay using a set of primers FP: GGATCG CCC AC CACG ATTAC TACC and RP: ACTG CCT CACACAACC TCATC TCC [14]. The utilized isolated MDV was titrated in duck embryo fibroblast (DEF) according to Thornton [15], and used for the experimental infection of chicks of different breeds. Eight different native breeds namely (Gimmizah, Sinai, Dandarawi, Fayoumi, Golden Montazah, Matrouh, Beheri, Dokki) in addition to SPF Lohmann, and High Line of 1 day old which carry *ChB6* gene and group of chicken (Bovans) which lacks *ChB6* gene of 1 day old and all have no maternal antibodies against MDV [16] were used for the experiment.

Eight hundred and twenty-five of 1-day-old chicks were divided into 11 different groups each group of 75 chicks, and all groups were experimentally infected subcutaneously with 0.5 mL MDV separately 1×10^3^ PFU [17]. Spleen samples were collected from all infected groups at 20^th^, 25^th^, 30^th^, 35^th^, and 40^th^ weeks post-infection from different groups and tested by PCR assay for the detection of MDV [18]. Furthermore, at 40^th^ week post-infection, tumorized spleen sample of Bovans breed was collected and prepared for examination by transmission electron microscope (TEM) to confirm the presence of MDV [19].

## Results

**Figure-1:**
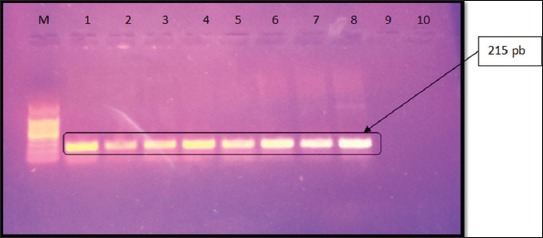
Demonstrate electrophoresis of the amplified products for detection of the *ChB6* gene. M represents the 100 bp molecule PCR marker. Lanes 1-8 represent the specific PCR product of positive breeds at the correct expected size for *ChB6 (*215 bp). Lanes 9 and 10 were negative.

The PCR amplification for the detection of *ChB6* gene in these 14 chicken breeds concluded that the *ChB6* gene was found in 10 breeds (A-J) as they showed positive amplification for *ChB6* gene at 215 bp, while the rest four breeds (K-N) were negative to the *ChB6* gene (Table-1 and Figure-1).

**Table-1:** The detection of *ChB6* gene in different 14 chicken breeds.

Group No	Code number of breeds	Name of the breeds	Source of the breeds	Number of the chicken in each breed	Detection of *ChB6* gene
1	A	Dokki	Elazzab project in fayoum	30	Positive
2	B	Gimmizah	Elazzab project in fayoum	30	Positive
3	C	Sinai	Elazzab project in fayoum	30	Positive
4	D	Dandarawi	Elazzab project in fayoum	30	Positive
5	E	Fayoumi	Elazzab project in fayoum	30	Positive
6	F	Golden Montazah	Elazzab project in fayoum	30	Positive
7	G	Matrouh	Elazzab project in fayoum	30	Positive
8	H	SPF Lohmman	Koom osheem in Fayoum	30	Positive
9	I	Beheri	Elazzab project in fayoum	30	Positive
10	J	Layer breed High Line		30	Positive
11	K	Layer breed bovans		30	Negative
12	L	Mandarah	Elazzab project in Fayoum	30	Negative
13	M	Inshas	Elazzab project in fayoum	30	Negative
14	N	Roodiland	Elazzab project in fayoum	30	Negative

**Figure-2:**
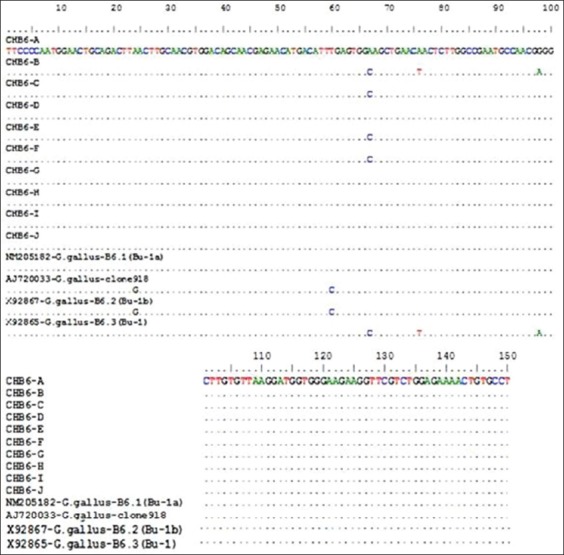
Nucleotide (nt) alignment of *ChB6* gene using Clustal format alignment by BioEdit (V6.5), *ChB6* gene sequence for the alignment of nt.

**Figure-3:**
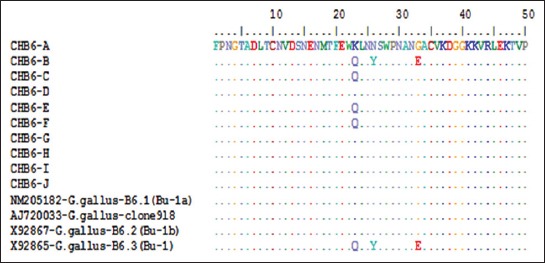
Alignment of *ChB6* gene using Clustal format alignment by BioEdit (V6.5), *ChB6* gene sequence for the alignment of amino acid in the 10 positive groups and other similar sequences from GenBank.

**Figure-4:**
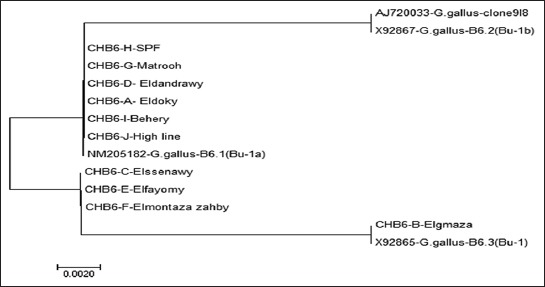
Phylogenetic tree of nucleotide sequences of *ChB6* gene in 10 different positive breeds and other related breeds from GenBank.

The genomic sequencing (nt and amino acid analysis) of chicken *ChB6* detected the presence of *ChB6* gene in the 10 positive chicken breeds and other similar gene sequences from GenBank. Each set row is a total of 150 nt. Dots indicate the position where the sequence is identical to the consensus (Figures-2-4 and Table-2).

**Table-2:** Identity and diversity percentage of the *ChB6* gene compared to some reference genes.

	Seq ID	1	2	3	4	5	6	7	8	9	10	11	12	13	14
1	ChB6-A		98	99	100	99	99	100	100	100	100	100	99	99	98
2	ChB6-B	98		99	98	99	99	98	98	98	98	98	97	97	100
3	ChB6-C	99	99		99	100	100	99	99	99	99	99	98	98	99
4	ChB6-D	100	98	99		99	99	100	100	100	100	100	99	99	98
5	ChB6-E	99	99	100	99		100	99	99	99	99	99	98	98	99
6	ChB6-F	99	99	100	99	100		99	99	99	99	99	98	98	99
7	ChB6-G	100	98	99	100	99	99		100	100	100	100	99	99	98
8	ChB6-H	100	98	99	100	99	99	100		100	100	100	99	99	98
9	ChB6-I	100	98	99	100	99	99	100	100		100	100	99	99	98
10	ChB6-J	100	98	99	100	99	99	100	100	100		100	99	99	98
11	NM205182-*G. gallus*-B6.1 (Bu-1a)	100	98	99	100	99	99	100	100	100	100		99	99	98
12	AJ720033-*G. gallus*-clone918	99	97	98	99	98	98	99	99	99	99	99		100	97
13	X92867-*G. gallus*-B6.2 (Bu-1b)	99	97	98	99	98	98	99	99	99	99	99	100		97
14	X92865-*G. gallus*-B6.3 (Bu-1)	98	100	99	98	99	99	98	98	98	98	98	97	97	

Gallus gallus=G. gallus

The nt sequence showed identity range from 98% to 100% to the *ChB6* gene in 10 different positive breeds and other similar sequences from GenBank. Each set row is a total of 50 amino acids. Dots indicate the position where the sequence is identical to the consensus.

### Experimental infection trial

**Figure-5:**
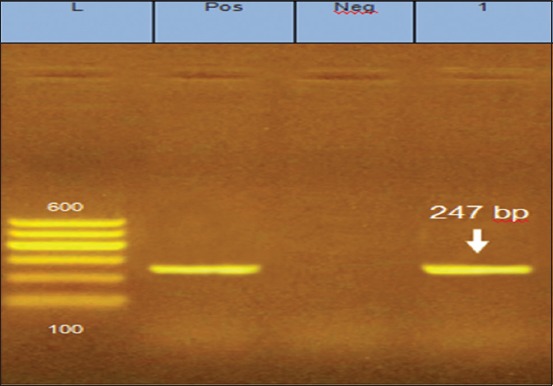
Polymerase chain reaction for the tissue samples (liver and spleen) infected by Marek’s disease virus. L for 100 bp ladder. Pos - Positive control. Neg - negative control. 1 sample.

The isolated virus was obtained from liver and spleen samples of some Kaloubia layer farms which revealed positive amplification of MDV by PCR assay to be ready for experimental infection (Figure-5).

**Figure-6:**
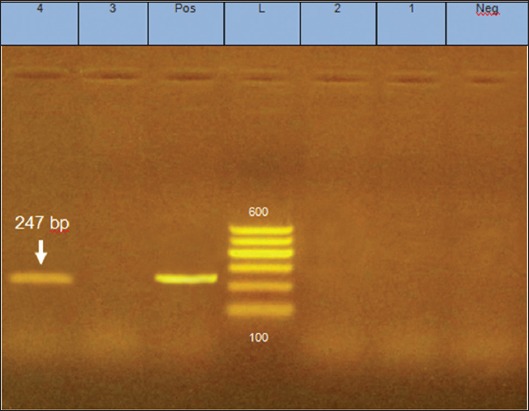
Polymerase chain reaction for the spleen of experimentally infected birds by Marek’s disease virus. L - Ladder. Lanes 1-3 - negative amplification of samples (A, C, and E). Lane 4 - positive amplification of sample (K). Lane Pos - positive control.

After infection of DEF by different dilutions of the isolated virus, the titer was 10^4^ PFU/0.5 ml. The collected spleen samples at 25, 30, 35, and 40 weeks post-infection were detected by PCR assay, the groups (A-J) revealed negative amplification of 247 bp of MDV gene, whereas spleen samples collected from groups (K) revealed positive amplification at 247 bp of MDV gene (Figure-6).

### Electron microscope characterization of reisolated MDV

**Figure-7:**
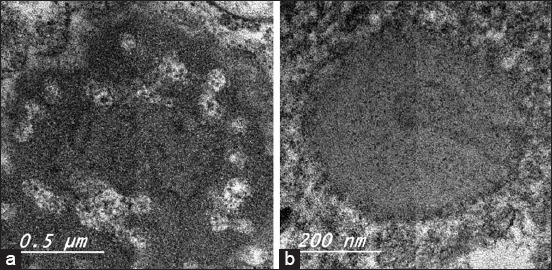
(a-b) Transmission electron microscopy of susceptible breed spleen.

The result of TEM indicated the presence of MDV viral particles in the cytoplasm of lymphocytes and macrophages in spleen sample of 40 weeks post-infection in susceptible breed having no *ChB6* gene (Bovans) (Figure-7).

## Discussion

The major goal in the current animal breeding industry is to improve animal health [20]. By genetic control of resistance to pathogens, we can reduce the disease-related costs and losses in addition to improvement of the immune capacity of animals. The complex immune system of poultry provides an opportunity for investigating polygenic regulation of immune response in chickens [21].

The *ChB6* gene has been proposed as a candidate gene in regulating the development of B-cell [5]. The *ChB6* gene is expressed on the B-cell precursor in the chicken embryo and some macrophages [22,23]. It was previously reported that *ChB6* is associated with the expression level of major histocompatibility complex (MHC) Class II, regression of *Rous sarcoma*, and resistant to MD [6,24,25]. Morever, also, *ChB6* is mapped on chromosome [26].

The chicken B-cell marker (*ChB6*) gene is considered as a candidate gene in the regulation of B-cell development [6]. *ChB6* was associated with the resistance to MD, regression of *R. sarcoma*, and expression level of MHC Class II [6,24]. In the present study, we tested 14 breeds for the presence of *ChB6* gene to investigate the resistance of such breeds to MDV-induced tumors. No reports have been published to document the resistance of these breeds to MDV.Results have shown that *ChB6* gene was detected in 10 native breeds (Gimmizah, Sinai, DandarawiFayoumi, Golden Montazah Matrouh Beheri Dokki), also in SPF Lohmann, and High Line breeds; no reports have been published the occurrence of tumor in such breed, while the other four tested breeds (Bovans, Elmandra, Anshase, and Roodiland) were negative to the *ChB6* gene.

Amplification of 215 bp fragment in PCR reaction confirms the correct expected size of the *ChB6* gene [9]. Such PCR assay was previously reported by Tregaskes *et al*. [6] and O’Laughlin [27], and the results of the present study confirm the specificity of utilized primers in the PCR reaction. Sequencing of the amplified product of *ChB6* gene revealed that there is a high similarity between the 10 positive chicken breeds (Gimmizah, Sinai, Dandarawi, Fayoumi, Golden Montazah, Matrouh, Beheri Dokki, SPF Lohmann, and High Line).

As previously reported, the similarity in the *ChB6* in different breeds was high [6,10]. Such similarity was also reported when we search on the reported sequences in GenBank [9,27]. The homology percentage ranged between 98% and 100% in the 10 positive breeds confirms the conservation of *ChB6* gene. Greatest identity in the hypervariable region analyzed in the obtained sequences has been noticed. Such identity was reported in all 10 positive breeds and also in other sequences found in GenBank. Sequence analysis of the 150 nt fragments of the amplified *ChB6* gene was obtained and was submitted to NCBI GenBank under Accession Number AJ720033-*Gallus gallus*-clone918, X92867-*G. gallus*-B6.2 (Bu-1b), X92865-*G. gallus*-B6.3 (Bu-1), and NM205182-*G. gallus*-B6.1 (Bu-1a). The sequence of *ChB6* gene from the 10 positive breeds was aligned. Nt sequence analysis revealed the grate identity between Matrouh, Beheri, Dokki, SPF Lohmann, Dandarawi, High Line, and NM205182-*G. gallus*-B6.1 (Bu-1a) and identity between Sinai, Fayoumi, and Golden Montazah while there is identity between Gimmizah and X92865-*G. gallus*-B6.3 (Bu-1) and identity between AJ720033-*G. gallus*-clone 918 and X92867-*G. gallus*-B6.2 (Bu-1b).

The amino acid sequence showed identity range from 94% to 100% to the *ChB6* gene in 10 different positive breeds. The hypervariable region (150 nt) contains the most informative genetic data regarding the *ChB6* gene, it was chosen for sequence analysis to characterize the sequence of *ChB6* in 10 different positive chicken groups (Gimmizah, Sinai, Dandarawi, Fayoumi, Golden Montazah, Matrouh, Beheri, Dokki SPF Lohmann, and High Line) and the relation between them comparing with similar sequences from GenBank. In this study, a comparative alignment and phylogenetic analysis of 150 nt fragments of the amplified *ChB6* gene revealed greatest identity. Characterization of the challenge virus isolated from field samples was confirmed by PCR [14,28]. We used 10^4^ PFU of the isolated virus for challenge as reported by Thornton [15].

Trial for the isolation and identification of MDV from spleen collected from challenged birds at 25, 30, 35, and 40 weeks’ post-infection by PCR assay showed negative results in the breeds containing *ChB6* gene while another breed (Bovans) which revealed negative for having *ChB6* gene shows positive.

Isolation from spleen as previously reported is the organ of choicer for reisolation [18]. TEM indicated the presence of MDV viral particles in the spleen of Bovans breed to confirm the PCR results. Characteristic MDV viral particles were observed like previously reported [19].

## Conclusion

The study confirms the relationship between the presence of *ChB6* gene in our native breed and the absence of tumors. This will need further investigations to enhance the breeding program of such breed in Egypt.

## Authors’ Contributions

HAH, MME, and MAS designed and carried out the main research work. The research was carried by HAS, MME, and HAH. The manuscript was written by HAS and HAH. All authors read and approved the final manuscript.
